# *Antioxidants*: Looking Forward after a Decade

**DOI:** 10.3390/antiox10121992

**Published:** 2021-12-14

**Authors:** Stanley T. Omaye

**Affiliations:** Department of Nutrition and Director, Environmental Sciences and Health Graduate Program, University of Nevada, Reno, NV 89557, USA; omaye@unr.edu; Tel.: +1-7750784-6447

As our journal, *Antioxidants*, celebrates its tenth year, I want to express my gratitude to our publisher, MDPI, the editorial staff, our editors and reviewers, and the many authors for making it possible for *Antioxidants* to become a respective premier journal. *Antioxidants* has met all the hallmarks of excellence that distinguish it from our field’s top-tier journals, and I am humbled by the small part I played in that achievement. When my esteemed colleague Dr. Nabil Elsayed and I were approached to lead the journal, we were highly optimistic about its potential success [1]. It has been rewarding to see *Antioxidants* mature as an academic journal.

Notable accomplishments [2] include: being indexed within Scopus, SCIE (Web of Science), PubMed, PMC, FSTA, AGRICOLA, CAPlus/SciFinder, and many other databases. Our journal ranks 11 out of 143 (Q1) titles in Food Sciences and Technology; 60 out of 295 (Q1) in Biochemistry & Molecular Biology; and 6 out of 62 (Q1) titles in Medicinal Chemistry. Our Impact Factor was 6.313 for 2020, with a 5-Year Impact Factor of 6.648. These are significant achievements for a decade-old journal.

Dr. Elsayed and I speculated that [1] low to marginalized antioxidant status likely leads to specific populations experiencing adverse health outcomes. Although dietary fruits and vegetable nutrients still correlate well with health, the usefulness of supplements or pharmacologic doses of certain antioxidants was somewhat speculative. The challenge for this field was to mitigate the consequences of unchecked oxidative stress and not produce unforeseen cellular implications, i.e., pro-oxidant effects. During the past decade, our authors have provided far-reaching insights into many adverse health conditions that manifest through oxidative stress, such as chronic diseases, infectious pathogens, environmental pollutants, and various noxious chemicals. We have also witnessed the era of mitigating oxidative stress by a range of antioxidants found in natural products or developed/synthesized in the laboratory to target key molecular events in the oxidative process.

Therefore, we look forward to the next decade for what lies in the future in developing our understanding of oxidative stress and antioxidants. We will be continuing to strive to remain true to our journal’s aims and scope: (1) to provide an advanced forum for studies related to the science and technology of antioxidants; (2) to publish research papers, reviews and communications at the forefront of research. We aim to encourage scientists to publish their experimental and theoretical results in as much detail as possible. The scope of this journal provides an advanced forum for studies related to the science and technology of antioxidants, focusing on new insights and ideas on active species and processes of biological relevance, natural products, mechanisms of action, applications and uses. We look forward to the continued success of *Antioxidants* in the next decade.
